# Editorial: Letter-sound knowledge, reading, reading comprehension and full literacy

**DOI:** 10.3389/fpsyg.2023.1292457

**Published:** 2024-01-04

**Authors:** Heikki Lyytinen, Elena L. Grigorenko, Hermundur Sigmundsson

**Affiliations:** ^1^Department of Psychology, University of Jyväskylä, Jyväskylä, Finland; ^2^Department of Psychology, University of Houston, Houston, TX, United States; ^3^Department of Psychology, Norwegian University of Science and Technology, Trondheim, Norway

**Keywords:** reading, letter-sound knowledge, children, basic reading skill, full literacy, gender

This Research Topic was proposed to illustrate the steps of reading acquisition that any learner has to take to reach the goal of reading, reading for meaning or full literacy (FL), where reading is both enjoyable and is the major instrument of learning (Sigmundsson et al., 2022). The creation of this issue was motivated by the fact that education authorities appear not to realize the ongoing transformation of reading habits in the world. Boys especially seem not to be interested in using their time for leisure reading as much as they used to do earlier and in some contexts need more reading training/experience to learn to enjoy reading (Lyytinen and Louleli; Sigmundsson et al., 2017). This compromises their opportunity to take the steps needed to achieve FL and importantly, this aim, given only a few years ago, seems to not be happening anymore. This has been demonstrated by the continuous decline in international literacy assessment results around the world. To illustrate, Finland is one of the most literate countries. It is also one of the most “digitalized”, where the use of computer games is highly popular among boys. Lack of interest in leisure reading is a serious issue, as it has started affecting the school achievements of Finnish boys, as documented by an increasing gender difference with advantages for girls. It is noteworthy that boys face reading difficulties, although they have no cognitive limitations to explain the problem (Lyytinen and Louleli; Sigmundsson et al., 2017). They have sufficiently fluent and accurate basic reading skills: importantly, given the nature of the Finnish language and how it facilitates these basic skills, almost no first-graders fail in Finland.

School authorities tend to continue defining reading skills as a unitary phenomenon. Yet the world in which we are now training children to acquire reading skills has changed. Educators need to understand that reaching the goal of reading requires students to take two steps before FL is acquired. Accurate and fluent basic reading skill is not enough: leisure reading is paramount, so it can, as previously, elevate Finnish children to their top level in literacy acquisition. Sadly, there are almost no Finnish boys who read for pleasure now.

In low-income countries, such as countries in Sub-Saharan Africa, children have problems learning to store consistent letter-sound correspondences (Sampa et al., 2018) and, thus, the acquisition of basic reading skills is an issue. However, even if African children are trained to learn these skills, as we have demonstrated before (Jere-Folotiya et al., 2014; Ojanen et al., 2015) and documented that they can sound out different pronounceable sequences of letters, they still face the problem of learning to comprehend what they read. A potential reason might be that they do not have adequate reading material that they enjoy reading and enable a transition from reading enough to reach the goal of reading via leisure reading. Sadly, there are similar tendencies in middle- and low-income countries.

This Research Topic includes eight articles from researchers around the world. The first article is from Spain and investigates the cognitive correlates of word reading (González-Valenzuela et al.). The second article from Portugal focuses on how to effectively intervene to elevate decoding and spelling (that is, basic reading skills, BRS) (Sucena et al.). The third article from Thailand exclusively attended to the correlates of BRS (Thongseiratch et al.). Perhaps surprisingly, these manuscripts did not give any attention to the role of reading activity unfolding for the sake of reaching the goal of reading; importantly, both unavailability and interest in reading materials can compromise children's reading activity and thus reaching the ultimate goal of FL. The fourth contribution from China switches our attention and directs it to the meaning of the text by focusing on morphological awareness specific to the writing systems of China (Liu et al.). Yet, it looks at reading only at word level and does not reflect the issues associated with reaching the goal of reading. The fifth article from the Dominican Republic approaches the goal of reading, as the authors examine the precursors of reading comprehensiQn and emphasize the important role of spoken language skills as predictors of comprehension of written language (Sánchez-Vincitore et al.). The sixth article from Brazil attends to the visual aspects of learning the connections between letters and sounds, again limiting its interest in BRS-related issues (Resque et al.). The seventh article from China approaches comprehension-related issues by attending to collective picture book reading activities, which teach children to attend to meaning from the very beginning of their reading careers (Wang et al.). The eighth manuscript focuses on digital learning games and the importance of supporting the acquisition of FL with exciting materials that interest children (Lyytinen and Louleli).

The topic of this Research Topic was selected to bring to everyone's attention how important it is to take the goal of reading—learning to read to acquire knowledge by reading—more seriously than it has been done until now. It is essential to understand how the goal of reading can be reached via becoming first able to decode and recognize single words, but then via being willing to enjoy reading and start learning efficiently by reading.

The goal of reading is often forgotten in reading literature, where most research efforts have been focused only on the first step, i.e., on the acquisition of BRS (Sigmundsson et al., 2017). Today, we have to focus more on this goal, i.e., on the final step of acquiring FL. FL has to be a priority when building BRS, which UNESCO is documenting. Only FL matters when we observe learning from the schoolbooks.

International studies such as OECD's Programme for International Student Assessments (PISA) and PIRLS (Progress in International Reading Literacy Study), both show the levels of reading comprehension, assessed twice, in fourth grade and at the age of 15. Both of these have revealed very disturbing results, especially for Finland. The same may be observed soon in many other countries. We have to take the situation seriously because The Finnish Education Evaluation Center (FINEEC) has already shown what anyone could expect—the decline in school achievements. FINEEC also observed that the highest correlation to this decline is seen in how much learners read outside school.

Data revealing the portion of children who acquire appropriate FL could be an important predictor for the wellbeing of people in other regions. One could expect that the elevation of the FL of people in poor countries would soon reduce the poverty in the world.

## Author contributions

HL: Project administration, Writing – original draft. EG: Project administration, Writing – original draft. HS: Project administration, Writing – original draft.
